# Can Functional Magnetic Resonance Imaging Generate Valid Clinical Neuroimaging Reports?

**DOI:** 10.3389/fneur.2017.00237

**Published:** 2017-06-14

**Authors:** Roland Beisteiner

**Affiliations:** ^1^Study Group Clinical fMRI, High Field MR Center, Department of Neurology, Medical University of Vienna, Vienna, Austria

**Keywords:** functional magnetic resonance imaging, patient, presurgical mapping, brain mapping, methodology in clinical research

A highly critical issue for applied neuroimaging in neurology—and particularly for functional neuroimaging—concerns the question of validity of the final clinical result. Within a clinical context, the question of “validity” often equals the question of “instantaneous repeatability,” because clinical functional neuroimaging is done within a specific pathophysiological framework. Here, not only every brain is different but also every pathology is different, and most importantly, individual pathological brains may rapidly change in short time.

Within the brain mapping community, the problem of validity and repeatability of functional neuroimaging results has recently become a major issue. In 2016, the Committee on Best Practice in Data Analysis and Sharing from the Organization for Human Brain Mapping (OHBM) created recommendations for replicable research in neuroimaging, focused on magnetic resonance imaging and functional magnetic resonance imaging (fMRI). Here, “replication” is defined as “Independent researchers use independent data and … methods to arrive at the same original conclusion.” “Repeatability” is defined as repeated investigations performed “with the same method on identical test/measurement items in the same test or measuring facility by the same operator using the same equipment within short intervals of time” (ISO 3534-2:2006 3.3.5). An intermediate position between replication and repeatability is defined for “reproducibility”: repeated investigations performed “with the same method on identical test/measurement items in different test or measurement facilities with different operators using different equipment” (ISO 3534-2:2006 3.3.10). Further definitions vary depending on the focus, be it the “measurement stability,” the “analytical stability,” or the “generalizability” over subjects, labs, methods, or populations.

The whole discussion was recently fueled by an PNAS article published by Eklund et al. (1), which claims that certain results achieved with widely used fMRI software packages may generate false-positive results, i.e., show brain activation where is none. More specifically, when looking at activation clusters defined by the software as being significant (clusterwise inference), the probability of a false-positive brain activation is not 5% but up to 70%. This was true for group as well as single subject data (2). The reason lies in an “imperfect” model assumption about the distribution of the spatial autocorrelation of functional signals over the brain. A squared exponential distribution was assumed but found not to be correct for the empirical data. This article received heavy attention and discussion in scientific and public media and a major Austrian newspaper titled “Doubts about thousands of brain research studies.” A recent PubMed analysis indicates already 69 publications citing the Eklund work. Critical comments by Cox et al. (3)—with focusing on the AFNI software results—criticize the authors for “their emphasis on reporting the single worst result from thousands of simulation cases,” which “greatly exaggerated the scale of the problem.” Other groups extended the work. With regard to the fact that “replicability of individual studies is an acknowledged limitation,” Eickhoff et al. (4) suggest that “Coordinate-based meta-analysis offers a practical solution to this limitation.” They claim that meta-analyses allow “filtering and consolidating the enormous corpus of functional and structural neuroimaging results” but also describe “errors in multiple-comparison corrections” in GingerALE, a software package for coordinate-based meta-analysis. One of their goals is to “exemplify and promote an open approach to error management.” More generally and probably also triggered by the Eklund paper, Nissen et al. (5) discuss the current situation that “Science is facing a ‘replication crisis’.” They focus on the publicability of negative results and model “the community’s confidence in a claim as a Markov process with successive published results shifting the degree of belief.” Important findings are, that “unless a sufficient fraction of negative results are published, false claims frequently can become canonized as fact” and “Should negative results become easier to publish … true and false claims would be more readily distinguished.”

As a consequence of this discussion, public skepticism about the validity of clinical functional neuroimaging arose. At first sight, this seems to be really bad news for clinicians. However, at closer inspection, it turns out that particularly the clinical neuroimaging community has already long been aware of the problems with standard (“black box”) analyses of functional data recorded from compromised patients with largely variable pathological brains. Quite evidently, methodological assumptions as developed for healthy subjects and implemented in standard software packages may not always be valid for distorted and physiologically altered brains. There are specific problems for clinical populations and particularly for defining the functional status of an individual brain (as opposed to a “group brain” in group studies). With task-based fMRI—the most important clinical application—the major problems may be categorized in “patient problems” and “methodological problems.”

Critical patient problems concern:

– Patient compliance may change quickly and considerably.
– The patient may “change” from 1 day to the other (altered vigilance, effects of pathology and medication, mood changes—depression, exhaustion).
– The clinical state may “change” considerably from patient to patient (despite all having the same diagnosis). This is primarily due to location and extent of brain pathology and compliance capabilities.

Critical methodological problems concern:

– Selection of clinically adequate experimental paradigms (note paresis, neglect, aphasia).
– Performance control (particularly important in compromised patients).
– Restriction of head motion (in patients artifacts may be very large).
– Clarification of the signal source (microvascular versus remote large vessel effects).
– Large variability of the contrast to noise ratio from run to run.
– Errors with inter-image registration of brains with large pathologies.
– Effects of data smoothing, definition of adequate functional regions of interest, and definition of essential brain activations.
– Difficult data interpretation requires specific clinical fMRI expertise and independent validation of the local hardware and software performance (preferably with electrocortical stimulation).

All these problems have to be recognized and specific solutions have to be developed depending on the question at hand—generation of an individual functional diagnosis or performance of a clinical group study. To discuss such problems and define solutions, clinical functional neuroimagers have already assembled early (Austrian Society for fMRI,^1^ American Society of Functional Neuroradiology^2^) and just recently the Alpine Chapter from the OHBM^3^ was established with a dedicated focus on applied neuroimaging. Starting in the 1990s (6), this community published a considerable number of clinical methodological investigations focused on the improvement of individual patient results and including studies on replication, repeatability, and reproducibility [compare (7)]. Early examples comprise investigations on fMRI signal sources (8), clinical paradigms (9), reduction of head motion artifacts (10), and fMRI validation studies (11, 12). Of course the primary goal of this clinical research is improvement of the validity of the final clinical result. One of the suggested clinical procedures focuses particularly on instantaneous replicability as a measure of validity [Risk Map Technique (13–15); see Figure 1] with successful long-term clinical use. This procedure was developed for presurgical fMRI and minimizes methodological assumptions to stay as close to the original data as possible. This is done by avoiding data smoothing and normalization procedures and minimization of head motion artifacts by helmet fixation (avoiding artifacts instead of correcting them). It is interesting to note that in the Eklund et al. (1) analysis it was also the method with minimal assumptions (a non-parametric permutation), which was the only one that achieved correct (nominal) results. The two general ideas of the risk map technique are (a) to use voxel replicability as a criterion for functionally most important voxels (extracting activation foci = voxels with largest risk for a functional deficit when lesioned) and (b) to consider regional variability of brain conditions (e.g., close to tumor) by variation of the hemodynamic response functions (step function/HRF/variable onset latencies) and thresholds. The technique consists only of few steps, which can easily be realized by in house programming: (i) Record up to 20 short runs of the same task type to allow checking of repeatability. (ii) Define a reference function (e.g., step function with a latency of 1 TR). (iii) Calculate Pearson correlation *r* for every voxel and every run. (iv) Color code voxels according to their reliability at a given correlation threshold (e.g., *r* > 0.5): yellow voxels >75%, orange voxels >50%, red voxels >25% of runs need to be active. (v) Repeat (i)–(iv) with different reference functions (to our experience, a classical HRF and two step functions with different latencies are sufficient to evaluate most patients) and at different correlation thresholds (e.g., *r* > 0.2 to *r* > 0.9). The clinical fMRI expert performs a comprehensive evaluation of all functional maps with consideration of patient pathology, patient performance, and the distribution and level of artifacts [compare descriptions in Ref. (13, 15)]. The final clinical result is illustrated in Figure 1, and a typical interpretation would be: most reliable activation of the Wernicke area is found with a step function of 1 TR latency and shown at Pearson correlation *r* > 0.5. It is important to note that risk maps extract the most active voxel(s) within a given brain area and judgment of a “true” activation extent is not possible. However, due to the underlying neurophysiological principles [gradients of functional representations (16)], it is questionable whether “true” activation extents of fMRI activations can be defined with any technique.

**Figure 1:**
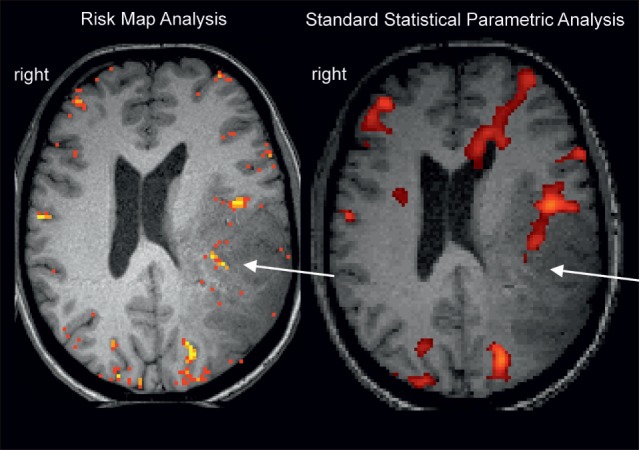
Example for a missing language activation (Wernicke activity, white arrow) with a “black box” standard analysis (right, SPM12 applying motion regression and smoothing, voxelwise inference FWE <0.05, standard *k* = 25) using an overt language design [described in Ref. (17)]. Wernicke activity is detectable with the clinical risk map analysis (left) based on activation replicability (yellow = most reliabel voxels). Patient with left temporoparietal tumor.

The importance to check individual patient data from various perspectives instead of relying on a “standard statistical significance value,” which may not correctly reflect the individual patients signal situation, has also been stressed by other authors [e.g., Ref. (18)]. Of course, clinical fMRI—as all other applied neuroimaging techniques—requires clinical fMRI expertise and particularly pathophysiological expertise to be able to conceptualize where to find what, depending on the pathologies of the given brain. One should be aware that full automatization is currently not possible neither for a comparatively simple analysis of a chest X-ray nor for applied neuroimaging. In a clinical context, error estimations still need to be supported by the fMRI expert and cannot be done by an algorithm alone. As a consequence, the international community started early with offering dedicated clinical methodological courses (compare http://oegfmrt.org or http://ohbmbrainmappingblog.com/blog/archives/12-2016). Meanwhile, there are enough methodological studies that enable an experienced clinical fMRI expert to safely judge the possibilities and limitations for a valid functional report in a given patient with his/her specific pathologies and compliance situation. Of course, this also requires adequate consideration of the local hard- and software. Therefore and particularly when considering the various validation studies, neither for patients nor for doctors there is a need to raise “doubts about clinical fMRI studies” but instead good reason to “keep calm and scan on.”^4^

## Author Contributions

The author confirms being the sole contributor of this work and approved it for publication.

## Conflict of Interest Statement

The author declares that the research was conducted in the absence of any commercial or financial relationships that could be construed as a potential conflict of interest.

## Footnotes

^1^http://oegfmrt.org.

^2^http://asfnr.org.

^3^http://humanbrainmapping.org.

^4^http://ohbmbrainmappingblog.com/blog/archives/07-2016.
